# Surface Decorations on Mixed Ionic and Electronic Conductors: Effects on Surface Potential, Defects, and the Oxygen Exchange Kinetics

**DOI:** 10.1021/acsami.3c03952

**Published:** 2023-05-22

**Authors:** Christoph Riedl, Matthäus Siebenhofer, Andreas Nenning, George E. Wilson, John Kilner, Christoph Rameshan, Andreas Limbeck, Alexander K. Opitz, Markus Kubicek, Juergen Fleig

**Affiliations:** † Institute of Chemical Technologies and Analytics, TU Wien, 1060 Vienna, Austria; ‡ Centre for Electrochemistry and Surface Technology, CEST, 2700 Wr. Neustadt, Austria; § Department of Materials, Imperial College, London SW7 2BX, United Kingdom; ∥ Chair of Physical Chemistry, Montanuniversität Leoben, 8700 Leoben, Austria

**Keywords:** surface decoration, mixed ionic electronic conductors, surface potential, defect chemistry, oxygen exchange, pulsed laser deposition

## Abstract

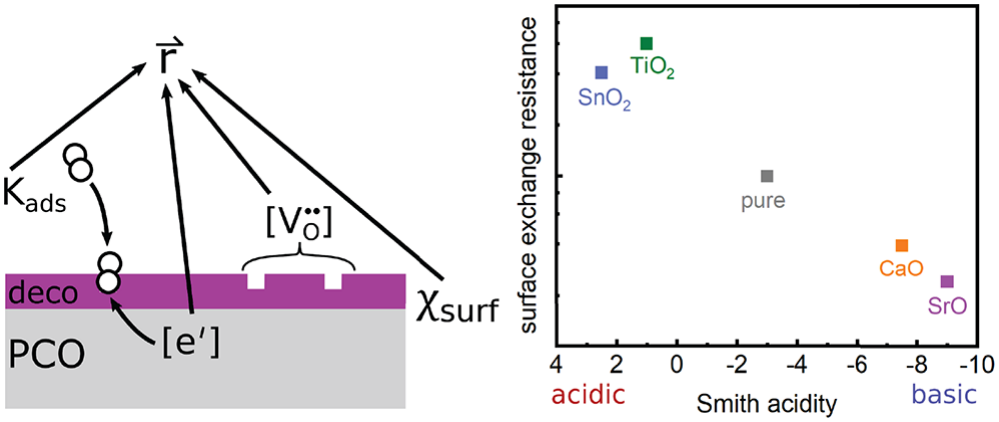

The oxygen exchange kinetics of epitaxial Pr_0.1_Ce_0.9_O_2−δ_ electrodes was modified by decoration with submonolayer amounts of different basic (SrO, CaO) and acidic (SnO_2_, TiO_2_) binary oxides. The oxygen exchange reaction (OER) rate and the total conductivity were measured by *in situ* PLD impedance spectroscopy (*i*-PLD), which allows to directly track changes of electrochemical properties after each deposited pulse of surface decoration. The surface chemistry of the electrodes was investigated by near-ambient pressure XPS measurements (NAP-XPS) at elevated temperatures and by low-energy ion scattering (LEIS). While a significant alteration of the OER rate was observed after decoration with binary oxides, the pO_2_ dependence of the surface exchange resistance and its activation energy were not affected, suggesting that surface decorations do not alter the fundamental OER mechanism. Furthermore, the total conductivity of the thin films does not change upon decoration, indicating that defect concentration changes are limited to the surface layer. This is confirmed by NAP-XPS measurements which find only minor changes of the Pr-oxidation state upon decoration. NAP-XPS was further employed to investigate changes of the surface potential step on decorated surfaces. From a mechanistic point of view, our results indicate a correlation between the surface potential and the altered oxygen exchange activity. Oxidic decorations induce a surface charge which depends on their acidity (acidic oxides lead to a negative surface charge), affecting surface defect concentrations, any existing surface potential step, potentially adsorption dynamics, and consequently also the OER kinetics.

## Introduction

Solid oxide fuel cells (SOFCs) and solid oxide electrolysis cells (SOECs) are among the most promising technologies for renewable power generation.^1−3^ Toward improving their general applicability, lowering the operation temperature to around 450–600 °C is one of the major challenges of current research activities. A lower temperature would bring several advantages like a reduced start-up time, improved long-term stability of the cells and the possibility to use materials, which are cheaper in purchasing and processing.^4,5^ However, lowering the operation temperature is also challenging, as several important processes in the cells are thermally activated, leading to high resistive losses at lower temperatures. Hence, current research is often directed toward improving the catalytic activity of existing materials (e.g., by infiltration) or developing novel, highly active materials.^6−8^

While currently mostly La_1–*x*_Sr_*x*_MnO_3−δ_ (LSM)-based or La_1–*x*_Sr_*x*_Co_1–y_Fe_*y*_O_3−δ_ (LSCF)-based composite cathode materials are applied in SOFCs,^9^ several recent studies have investigated numerous other mixed ionic and electronic conducting (MIEC) electrodes regarding their applicability as SOFC cathodes.^10−19^ While in state-of-the-art SOFCs, porous electrodes are applied due to their large electrochemically active surface area,^9^ model studies are often conducted on thin film electrodes grown by pulsed laser deposition (PLD) on single crystalline electrolyte substrates, exhibiting a well-defined surface morphology and stoichiometry and facilitating a homogeneous polarization of the electrode. In order to efficiently improve electrodes, only an in-depth mechanistic understanding of the oxygen exchange reaction (OER) on the electrode surface can significantly support a targeted, knowledge driven development of “real-life” SOFC electrodes.

Recently, several studies investigated the OER kinetics on cathode materials in great detail. They revealed that the oxygen exchange kinetics strongly depends on defect concentrations (e.g., holes, electrons, oxygen vacancies), which are affected by the doping concentration of the respective electrode material, pO_2_, and temperature.^16,17,20,21^ Trying to optimize these OER kinetics, other studies in literature investigated multiple approaches, for example, decoration with platinum nanoparticles,^22^ surface treatment with H_2_O^23^ or microstructural optimization.^24^ In addition, several studies were conducted to address methods of improving the stability of SOFC electrodes and to investigate their degradation behavior.^10,25,26^

In a recent pioneering work of Nicollet et al., a systematic change of the oxygen exchange activity of porous Pr_0.1_Ce_0.9_O_2−δ_ (PCO10) electrodes after infiltration with binary oxides was reported.^27^ The acidity scale for binary oxides as proposed by Smith was found to be a sensitive descriptor for the OER activity.^28^ While basic oxides (e.g., Li_2_O, CaO) led to an increase of the oxygen exchange activity (i.e., decrease of the electrode polarization resistance), acidic oxides (e.g., CrO_3_, Al_2_O_3_) decreased the oxygen exchange activity. The effect of submonolayer surface decorations was previously also demonstrated on the perovskite-type La_0.6_Sr_0.4_CoO_3−δ_, further substantiating the correlation of surface acidity with the surface exchange kinetics.^29,30^

Building on these promising results, we present a study on the detailed effects of surface decorations, employing *in situ* PLD impedance spectroscopy (*i*-PLD), near-ambient pressure X-ray photo electron spectroscopy (NAP-XPS), and low-energy ion scattering (LEIS). The study is performed on PCO10, as the fluorite structure is less prone to surface segregation and decoration intermixing than perovskites and facilitates a better reproducibility of the results. Epitaxial PCO10 thin films were decorated with acidic (TiO_2_, SnO_2_) and basic (SrO, CaO) binary oxides and their catalytic performance and conductivity were characterized during stepwise decoration by *i*-PLD, which allows to directly measure electrochemical properties while growing and decorating the thin film.^20,29^ A special focus is laid on characterizing the surface chemistry of PCO10 by NAP-XPS after decoration with the respective binary oxides. Thereby, effects like oxidation state or surface potential changes can be correlated with oxygen exchange kinetics (especially the latter being rarely investigated in literature).^31^ These results give a detailed picture of the various different effects of surface decorations and greatly improve our understanding of mixed conducting oxide surfaces.

## Results and Discussion

### Oxygen Exchange Activity upon Decoration

The effect of surface decoration with binary oxides on the OER resistance was measured by repeatedly preparing and modifying pristine PCO10 surfaces and tracking the impedance response in real time. Sample structures and the procedure are shown in the “Methods” section. More specifically, a 50 nm PCO10 thin film was deposited on top of the well-conducting GDC|LSC|GDC multilayer (XRD measurements of epitaxial thin films are shown in the Supporting Information). This PCO10 electrode was decorated stepwise with 1.5 monolayers (ML) of the respective binary oxide and the change of the impedance was tracked by *i*-PLD measurements. After depositing 1.5 ML, the pO_2_ dependence of the surface exchange resistance and its activation energy were examined to assess potential effects of the decoration on the oxygen exchange mechanism. After each decoration, 20 nm of fresh PCO10 was deposited on top of the electrode to reset the surface exchange resistance to a state comparable to before the decoration. As oxygen diffusion through PCO10 is fast in our thin films, the polarization resistance of the electrode is purely surface-related. The applied multilayer technique guarantees excellent comparability of impedance results as all measurements of the four different decoration materials were conducted on the same sample and at the same parameters (temperature, pO_2_, laser fluence, active electrode surface).

**Figure 1:**
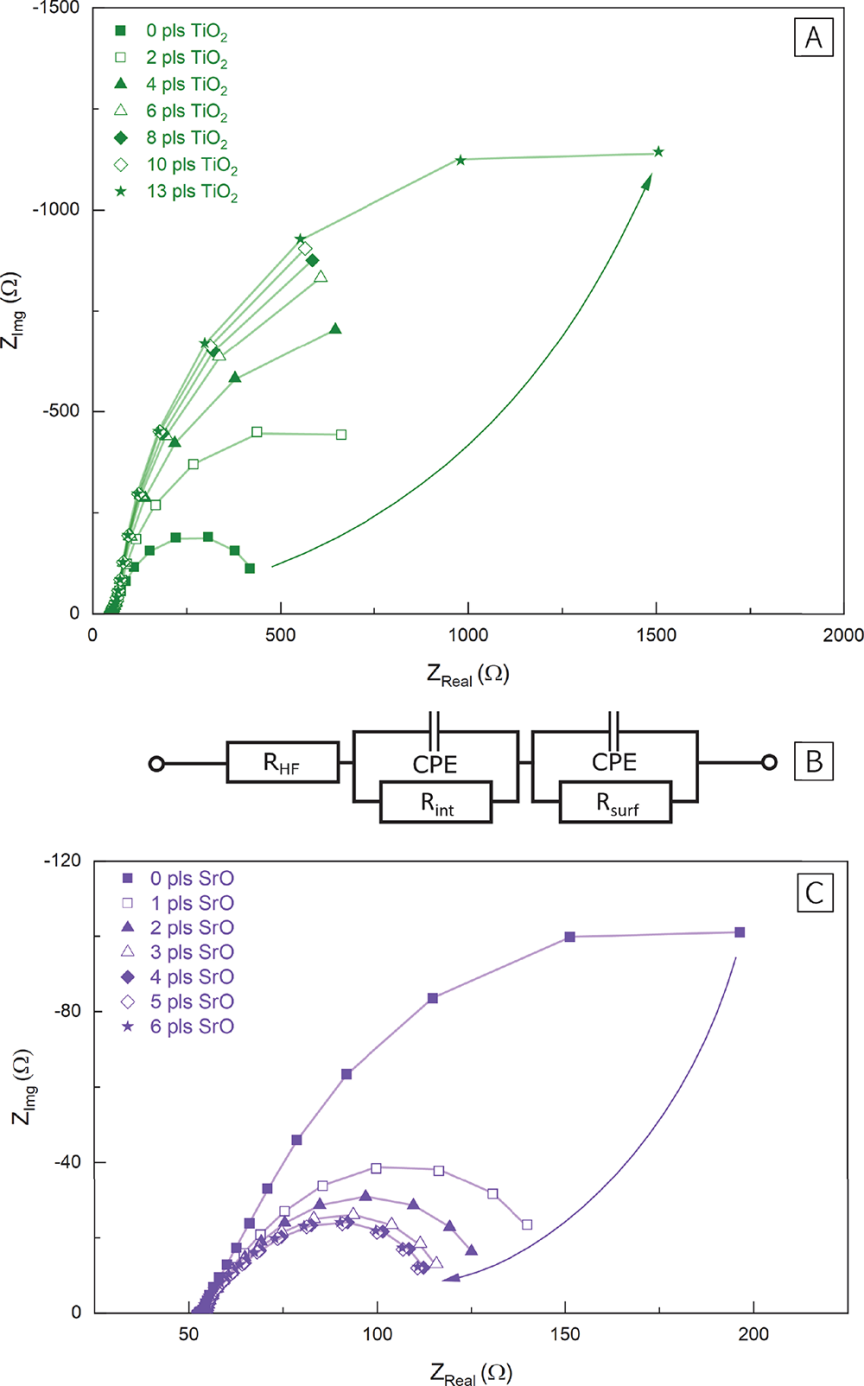
(A) Impedance spectra of a PCO10 electrode decorated with increasing amounts of TiO_2_. (B) Equivalent circuit used for fitting the spectra shown in (A) and (C). (C) Impedance spectra of a PCO10 electrode decorated with increasing amounts of SrO. Lines shown are fits conducted with the equivalent circuit in (B).

Figure 1 shows the change of the impedance spectra of a PCO10 electrode upon decoration with the acidic oxide TiO_2_ as well as with the basic oxide SrO. For fitting of the spectra, the equivalent circuit shown in Figure 1B was applied. The high-frequency intercept was assigned to ohmic resistances of wires and the Pt thin film grid (≈8 Ω) as well as the ionic conductivity of the YSZ substrate. The observed value (≈50 Ω) is in good accordance with the expected value for 600 °C.^32^ The main semicircle of the impedance spectra was fitted with an *R*∥CPE element, where the resistance, as several studies in literature have shown, can be assigned to the oxygen exchange on the surface of the electrode.^18,21,33,34^ This surface exchange resistance was further related to the active electrode surface area (PCO directly grown on YSZ), as past studies have shown the area above the Pt grid to be largely inactive for oxygen exchange on oxides with insufficient ionic in-plane conductivity.^33^ The shoulder in the mid-frequency range was fitted with an additional *R*∥CPE element and is mostly attributed to interfacial effects at the electrode–electrolyte junction, as well as double layer capacitances at interfaces.^35^ It is much smaller than the main arc and not further considered in this study.

**Figure 2:**
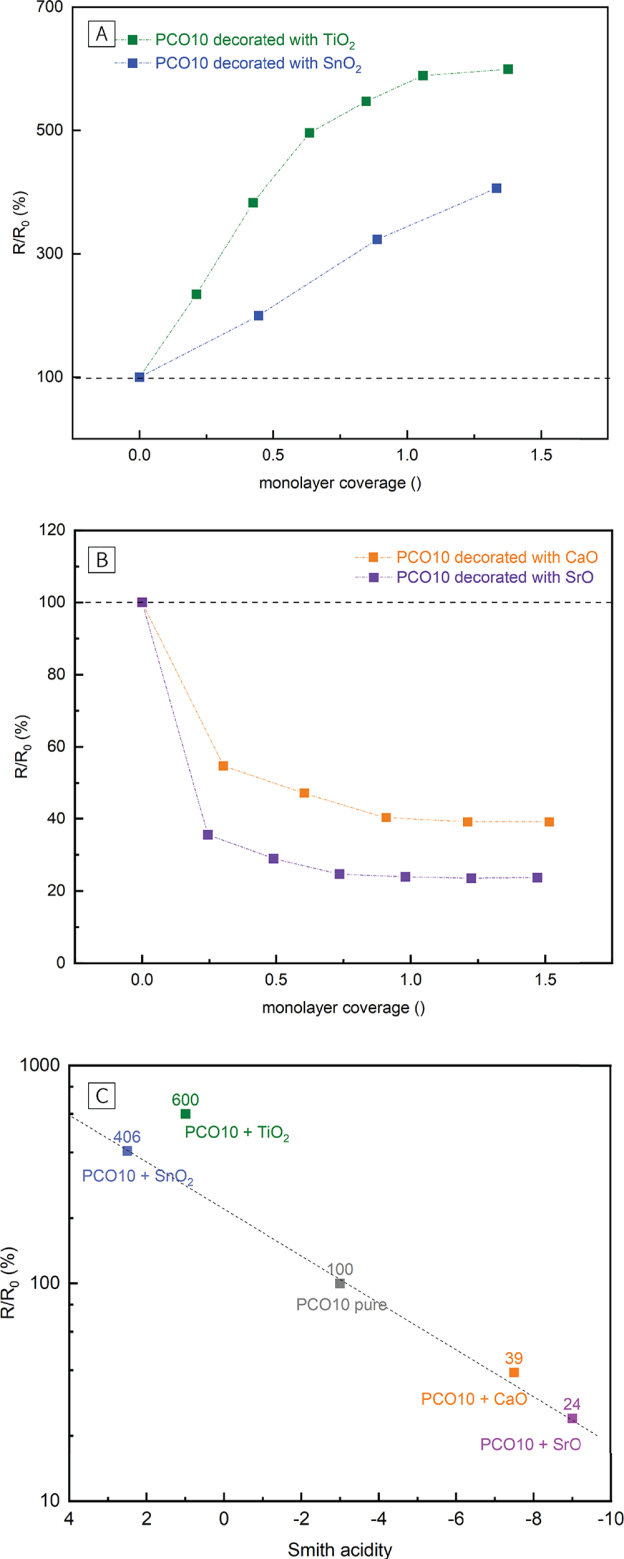
(A) Surface exchange resistance ratio compared to pure PCO10 after decoration with TiO_2_ and SnO_2_. (B) Surface exchange resistance ratio compared to pure PCO10 after decoration with SrO and CaO. All measurements were conducted at 600 °C and 0.04 mbar O_2_. (C) Surface exchange resistance ratio compared to pure PCO10 after decoration with the respective binary oxides plotted versus the Smith acidity.^28^

The overall change of the surface exchange resistance, normalized to pure PCO, is shown in Figure 2. The measurements reveal that acidic decoration leads to a substantial increase of the surface exchange resistance already with minor amounts of the binary oxide. For SnO_2_, the surface exchange resistance increased by 300% after the deposition of 1.5 ML of surface decoration. The effect of TiO_2_ surface decoration was even more pronounced with an increase of the surface exchange resistance by 500%.

For the two basic decorations CaO and SrO, the surface decoration had the opposite effect on the surface exchange resistance. While 1.5 ML of CaO reduced the surface exchange resistance of PCO10 to 40% of its original value, the improvement effect was even stronger with SrO, which reduced the surface exchange resistance to 25% of its original value. The beneficial effect of SrO is particularly interesting as most studies in literature reported Sr segregation in Sr-containing perovskites to be responsible for a strong performance decrease of the SOFC electrodes.^11,36^ However, recent studies have shown that gaseous species like CO_2_ or SO_2_ convert SrO to SrCO_3_ or SrSO_4_ in most testing setups and initially cause this degradation (but not during *i*-PLD).^30,37^ As has been suggested by Nicollet et al.,^27^ a clear correlation between the electrode performance and the Smith acidity is found (Figure 2C). Only the difference between SnO_2_ and TiO_2_ decorations is not in line with the corresponding acidity values. This shows that despite the significantly different preparation techniques (aqueous infiltration of porous PCO^27^ versus PLD-based decoration of thin films) the measurement results are in excellent accordance with previous investigations of similar surface decorations.

### pO_2_ and Temperature Dependence of the Surface Exchange Resistance of Pure and Decorated Surfaces

**Figure 3:**
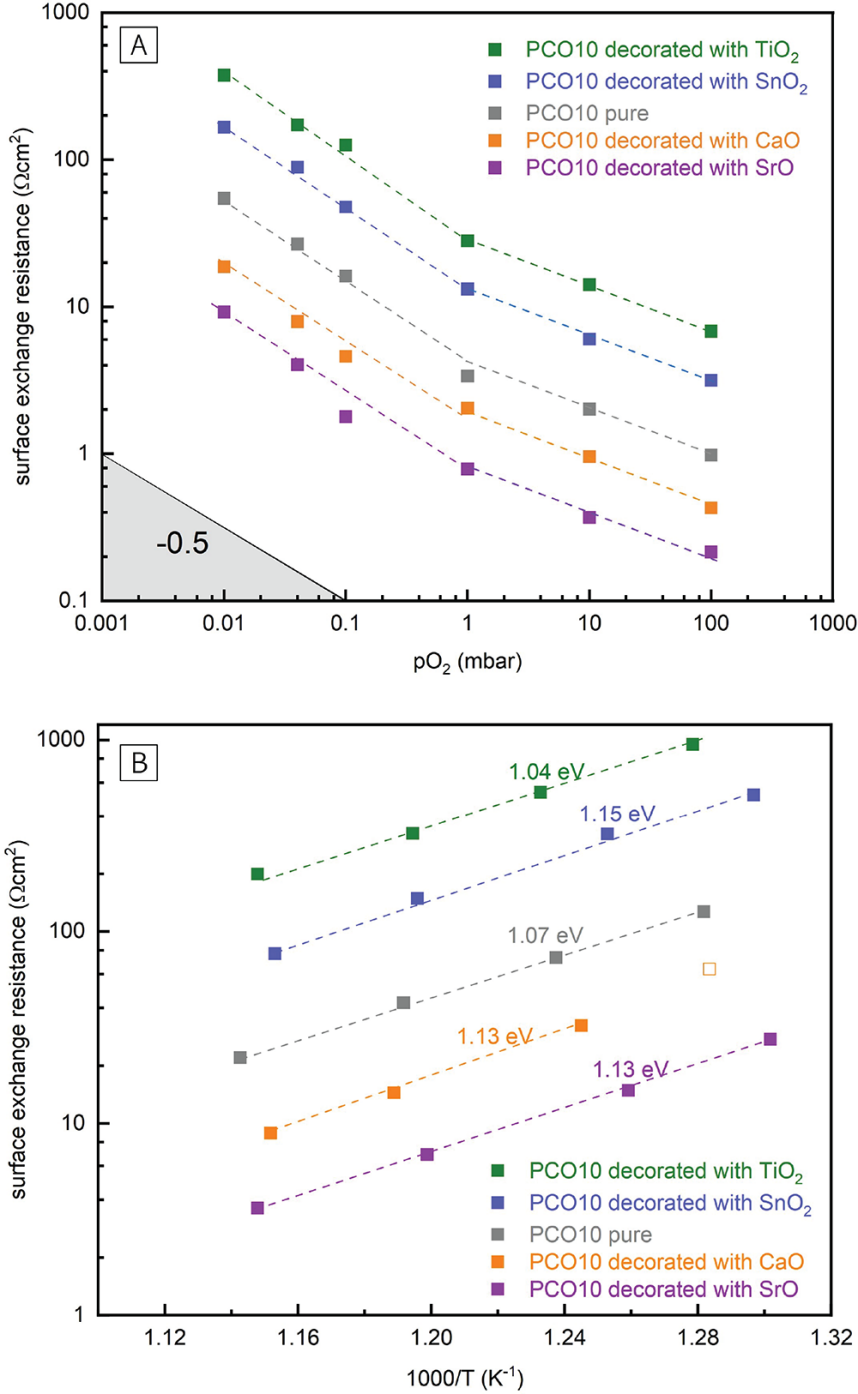
(A) pO_2_ dependence of the surface exchange resistance on pure and decorated PCO10 electrodes at 600 °C. (B) Determination of the activation energy on pure and decorated PCO10 electrodes at 0.04 mbar O_2_.

In order to assess a possible influence of surface decorations on the reaction mechanism, the pO_2_ dependence of the surface exchange resistance was investigated on pure and decorated PCO10 electrodes. In Figure 3A, the area-specific resistance is double-logarithmically plotted versus pO_2_, and it can be seen that all electrodes (pure and decorated) show the same pO_2_ dependence. While a steeper curve is found below 1 mbar O_2_, a flattening of the curve is observed at higher pO_2_. This dependence agrees very well with a recent *i*-PLD study in which the oxygen exchange mechanism was studied on different MIEC materials.^20^ As both pure and decorated PCO10 electrodes exhibit the same pO_2_ dependence, it is rather likely that the same oxygen exchange mechanism is active.

This is also supported by measurements of the activation energy on the respective electrodes. For determination of the activation energy, the polarization resistance was measured at different temperatures between 500 and 600 °C at 0.04 mbar O_2_ and plotted in an Arrhenius diagram (see Figure 3 B). The slopes reveal that no systematic alteration of the activation energy takes place during surface decoration with binary oxides. More specifically, while on pure PCO10 electrodes an activation energy of 1.07 eV was obtained, activation energies with acidic decorations were 1.04 eV (TiO_2_) and 1.15 eV (SnO_2_). Basic oxides lead to 1.13 eV (SrO) and 1.13 eV (CaO) with one data point being omitted for CaO. Thus, changes of the activation energy on decorated PCO10 electrodes were in the range of ±8% compared to the value measured on pure PCO10. No clear correlation between the Smith acidity and the change of the activation energy was found, supporting the conclusion that the decorations do not affect the mechanism of the oxygen exchange reaction itself. Nevertheless, it is worth mentioning that these activation energy changes are in the same order of magnitude as potential experimental errors.

### NAP-XPS Measurements of Pure and Decorated PCO10 Electrodes

In order to reveal how surface decoration with binary oxides affects the surface chemistry of PCO10 electrodes, near ambient pressure XPS (NAP-XPS) measurements were conducted. The main objectives of these measurements were to measure if surface decorations change the ratio of Pr^3+^ to Pr^4+^ in the upmost region of the PCO10 electrode and to investigate how surface decorations influence the surface potential of the PCO10 electrodes. For the NAP-XPS measurements, undecorated (“pure”) PCO10 electrodes were compared with PCO10 electrodes decorated with nominally 1.5 ML of either SnO_2_ or SrO. Samples were prepared in the *i*-PLD setup and then transferred to the NAP-XPS chamber. NAP-XPS measurements were carried out at 550 °C and 1 mbar O_2_, which was cleaned with a special gas purification column (Restek Super Clean Gas) to reduce sulfur contaminations in the measurement gas. Moreover, bias voltage was applied to electrochemically polarize the working electrode, thus modifying the internal oxygen chemical potential (i.e., the nominal oxygen partial pressure in the electrode). For mixed conductors with surface reaction limited kinetics such as PCO10, the equivalence of electrochemcial polarization and pO_2_ changes has been demonstrated in literature.^38,39^

**Figure 4:**
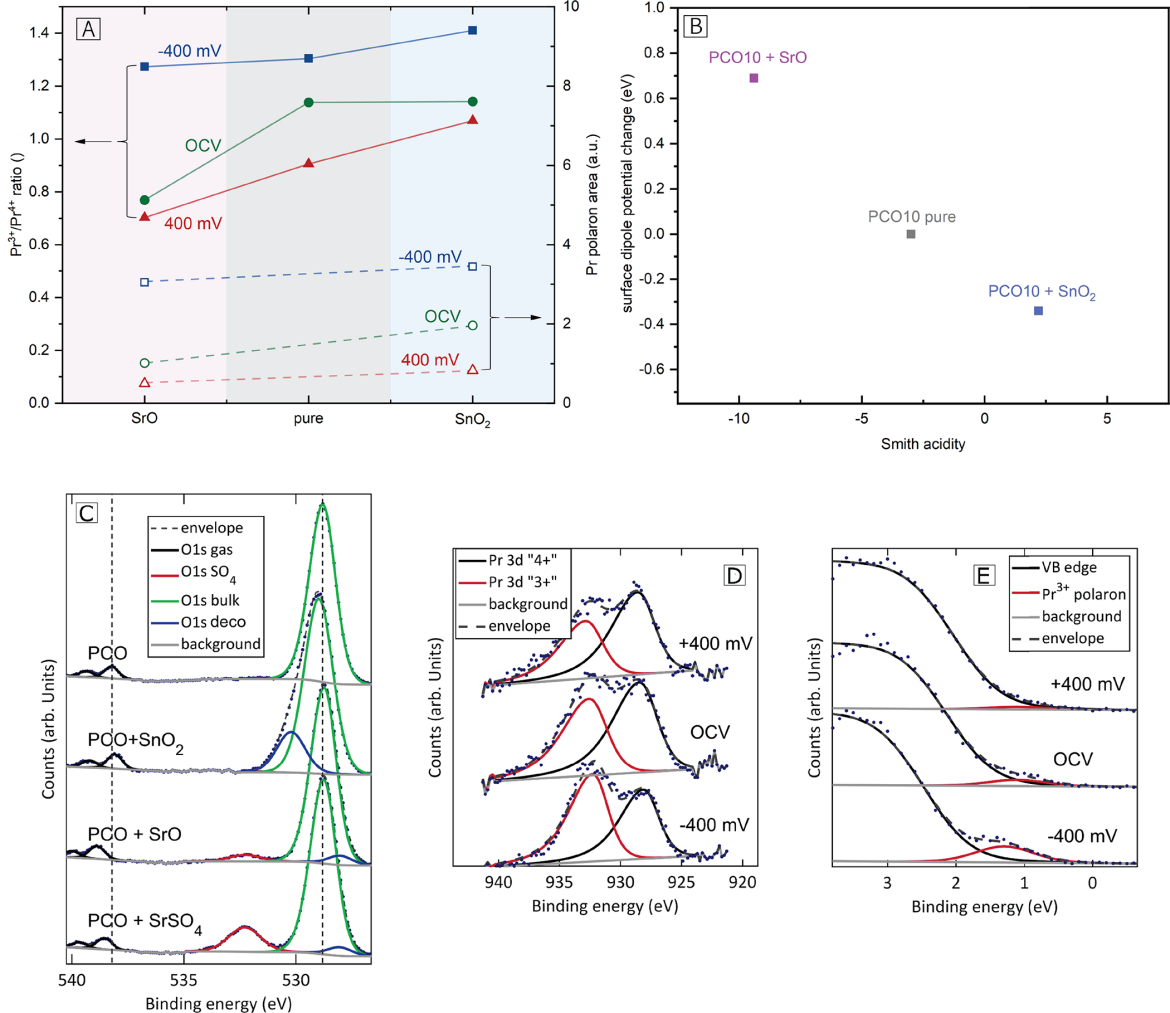
(A) Left *y*-axis: Pr^3+^/Pr^4+^ ratio on PCO10 pure, PCO10 decorated with 1.5 ML SrO or 1.5 ML SnO_2_ at OCV, under cathodic (−400 mV) and under anodic bias voltage (400 mV); right *y*-axis: Polaron area on PCO10 decorated with 1.5 ML SnO_2_ or 1.5 ML SrO at OCV, under cathodic (−400 mV) and under anodic bias voltage (400 mV). (B) Surface dipole potential change (BE(*O*_bulk_^PCO,pure^) – BE(*O*_2,gas_^PCO,deco^)) – (BE(*O*_bulk_^PCO,pure^) −BE(*O*_2,gas_^PCO,pure^)) for different decorations correlated with their Smith acidity. (C) Plot of the O 1s binding energy region on pure, SnO_2_ decorated, SrO decorated PCO10 electrodes, and PCO10 electrodes contaminated with SrSO_4_. (D) Pr oxidation states on PCO10 decorated with SrO at OCV and under different bias polarization. (E) Polaron area on PCO10 decorated with SrO at OCV and under different bias polarization.

As a possible mechanistic concept behind surface decorations, literature suggests that surface decorations lead to a band bending at the surface and thus increase (basic) or decrease (acidic) the electron concentration in the electrode subsurface and consequently the oxygen exchange activity of the decorated electrodes.^27^ In our XPS experiment, changes of the electron concentration in the subsurface are observable by changes of the Pr^3+^ concentration, which represent the majority electronic charge carrier in PCO10 at such conditions.^13^ In good accordance with literature,^40^ the Pr 3d_5/2_ peak is split into two components at 931 and 927 eV (Figure 4D). The reduction of Pr^4+^ to Pr^3+^ causes a change in the respective area ratio; however, both components are also present in extreme cases with 100% Pr^3+^ or Pr^4+^, making exact quantification nontrivial. In addition, polaronic Pr 4f electrons are visible in the valence band spectrum by a peak at ≈1.2 eV binding energy (BE) in the valence band spectra in Figure 4E, similar to the Ce^3+^ polaron feature that was already observed in reducing conditions.^41,42^

In Figure 4A, the ratio of Pr^3+^/Pr^4+^ on pure and decorated PCO10 electrodes under different bias polarization is shown. The results are in good agreement with the observed changes of the Pr^3+^ polaron area. As expected, the Pr^3+^ concentration increases with cathodic bias and decreases with anodic bias for all surface decorations; however, the results contradict the literature model,^27^ which predicts an increase in the electron concentration for SrO decorated PCO and a decrease for SnO_2_ decoration due to subsurface band bending. In contrast, we observed a slightly higher Pr^4+^ concentration for SrO decorated PCO at OCV, while PCO10 decorated with an acidic oxide (SnO_2_) and pure PCO10 showed the same ratio. Under anodic bias, PCO10 decorated with SrO showed a higher concentration of Pr^4+^ and a higher concentration of Pr^3+^ was found on PCO10 decorated with SnO_2_. In conclusion, the XPS measurements differ significantly from literature expectations as decoration with a basic oxide seems to slightly decrease the Pr^3+^ concentration in the upmost region of the thin film and vice versa for acidic decoration.

Changes of the surface dipole potential were investigated on pure and decorated PCO10 electrodes. For this purpose, the position of the oxygen gas phase peak with regard to the bulk O 1s binding energy was compared for pristine and decorated thin films. This difference is a measure for the potential step at the electrode surface (as elaborated in literature).^42^ In the notation chosen in this work, a more negative surface potential implies a more negatively charged surface. The binding energy difference found on PCO10 pure (9.45 eV) was set as the zero reference, and the resulting surface potential change was plotted in Figure 4B. The corresponding XPS spectra are shown in Figure 4C. Acidic decoration on a PCO10 electrode with 2 ML of SnO_2_ revealed a surface potential change of −0.34 eV, indicating a more negatively charged surface. In contrast, on PCO10 decorated with 2 ML of SrO (basic decoration) an increase of 0.69 eV was found. These results strongly suggest a systematic change of the surface potential depending on the acidity of the decorated oxide. This is further supported by the fact that sulfur contamination (with SO_2_ being a very acidic oxide) of the SrO decorated PCO10 over time (induced by trace impurities in the measurement gas) again reduced the investigated difference. In Figure 4B, the modified surface potential found on PCO10 electrodes is correlated with the Smith acidity of the decorations and a clear trend can be observed. Here it is also noteworthy that no decoration dependent changes in binding energies were found, although these would be expected if a conventional band bending occurs on the PCO10 surface. In conclusion, surface decorations alter the surface potential of PCO10 electrodes according to their relative acidity; however, a corresponding change of the Pr^3+^ concentration was not observed.

### Low-Energy Ion Scattering on Decorated PCO10

**Figure 5:**
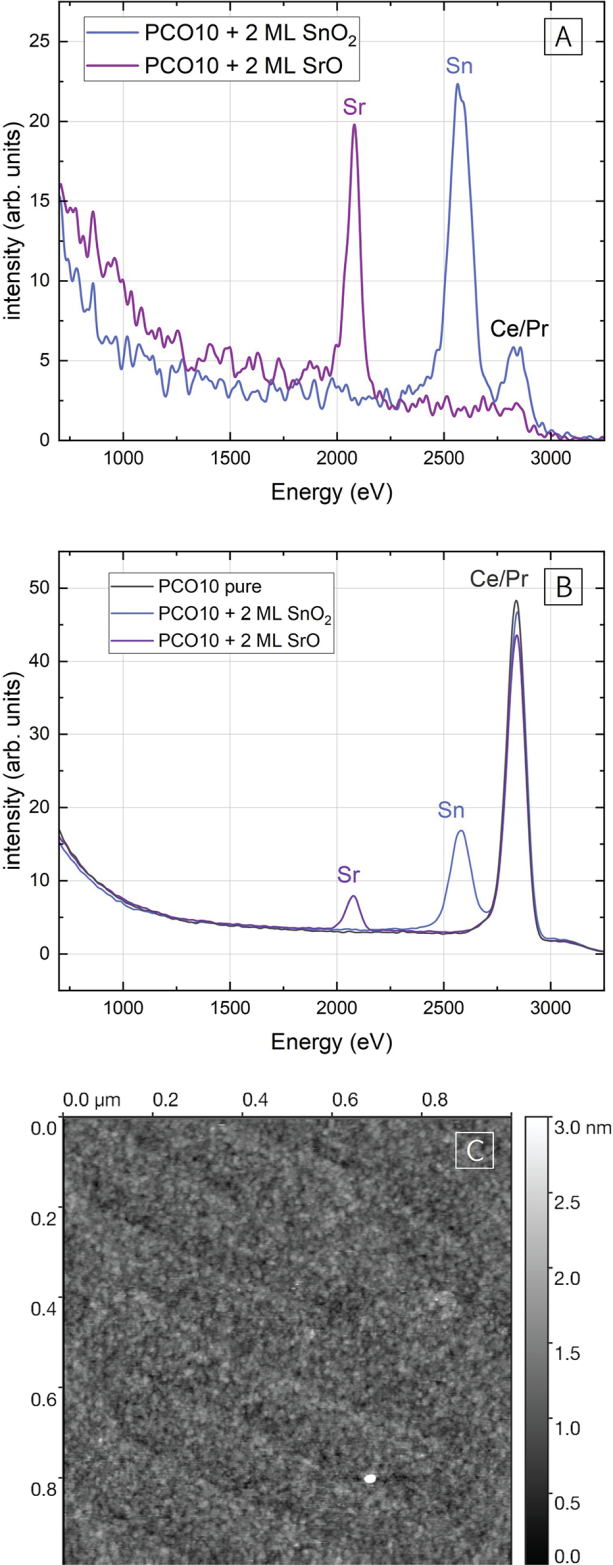
(A) LEIS spectra of the outermost surface of PCO10 thin films decorated with Sr and Sn. (B) Total of partial LEIS spectra recorded on a pure PCO10 thin film as well as Sr and Sn decorated PCO10 thin films. Decorations correspond to 2 ML each. (C) 1 × 1 μm^2^ AFM image of a PCO10 thin film grown on YSZ/GDC with 1.5 ML of SnO_2_ as top decoration.

As a complementary technique, which only probes the outermost atomic layer of the surface,^43,44^ LEIS measurements were performed on pure PCO10 thin films and on PCO10 thin films decorated with SrO and SnO_2_ (2 ML each). Figure 5 shows LEIS spectra of the three thin films. The shown total spectra (Figure 5B) are integrated over the first atomic layers of a depth profile measurement. The investigated surfaces are very clean (after reactive oxygen cleaning) and do not exhibit any signs of additional contaminations. They only exhibit a Ce/Pr signal (which overlaps due to the very similar mass of Ce and Pr) as well as the Sr/Sn signal of the decoration. From the spectra of the outermost surface (Figure 5A, for details on depth profiles, please refer to the Supporting Information), it becomes clear that the Pr/Ce cations on the surface are nearly completely covered by the decorated material (>90% of the total surface signal for both Sr and Sn, for SnO_2_ decoration, there is still some Pr/Ce visible, most probably due to local inconsistencies or different surface reconstructions during deposition of the sub-nm decoration layers).

To complement LEIS results on decorated PCO thin films, atomic force microscopy (AFM) was performed on an SnO_2_-decorated PCO10 thin film (Figure 5C). The PCO10 thin film was grown on YSZ with a GDC buffer layer and the atomic surface steps transfer through to the PCO10 surface. According to AFM and confirming LEIS results, the decoration does not agglomerate in larger clusters but seems to be finely dispersed on the PCO10 surface.

### *In Situ* Conductivity Measurements upon Decoration of Dense PCO Thin Films

**Figure 6:**
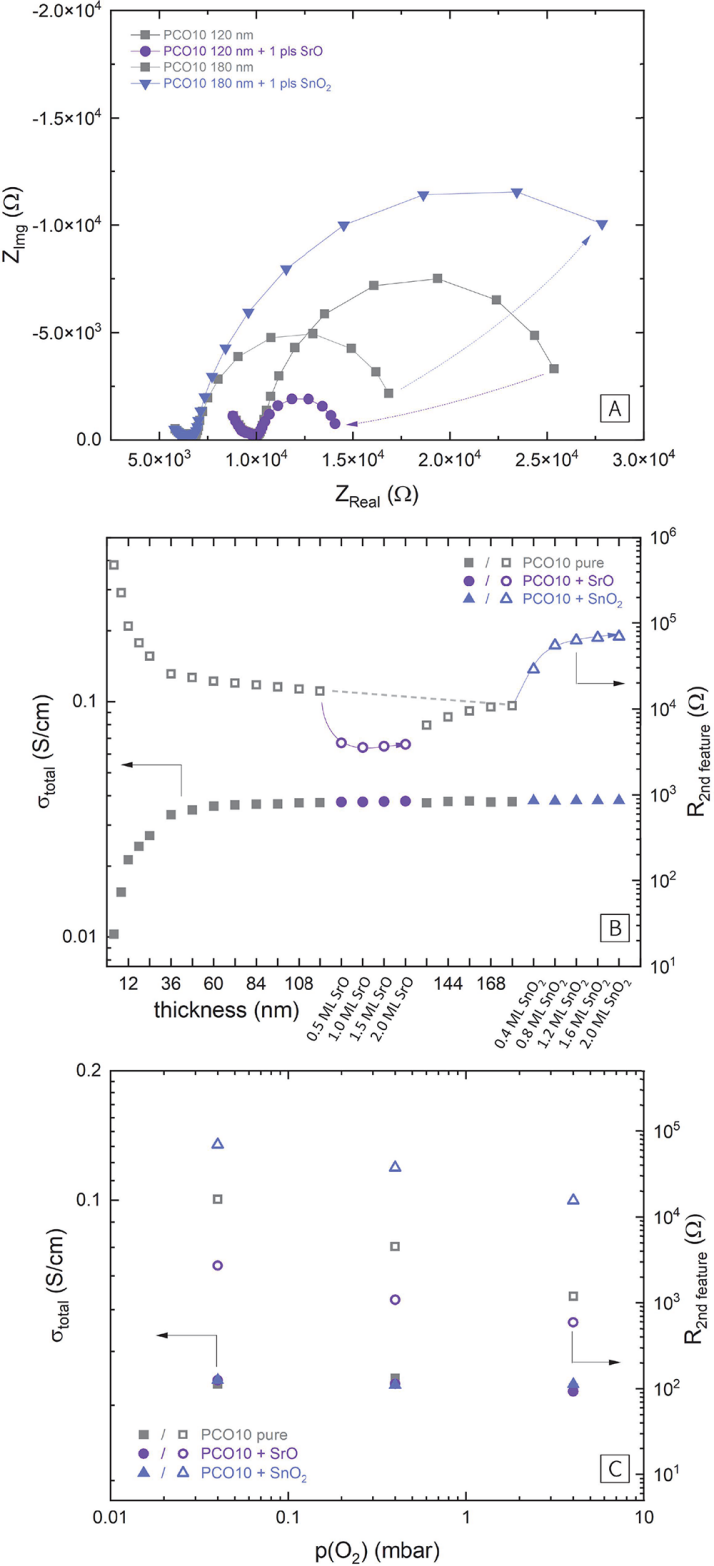
(A) Impedance spectra of pure PCO10 and the same surfaces decorated with 1 pls of SrO and SnO_2_. (B) Evolution of the total conductivity of the thin film and the ohmic contribution of the second impedance feature during the growth and decoration experiment. (C) Oxygen partial pressure dependence of the total conductivity and of the ohmic contribution of the second impedance feature.

To further investigate potentially changing defect concentrations, *i*-PLD was employed to determine the in-plane conductivity of a dense PCO10 thin film and its change upon decoration. For this, PCO thin films were epitaxially grown on insulating MgO substrates with a BZO/STO buffer layer as described in the experimental section. The in-plane impedance spectra measured between interdigitating finger electrodes show two well-separated features (Figure 6A): the end of a high frequency semicircle, which describes the total conductivity of the PCO thin film in parallel to the geometrical capacitance of the measurement configuration. The size of this arc steadily decreases with increasing film thickness (the extracted conductivity reaches a plateau, see Figure 6B). We suspect that the conductivity increase in early growth stages is due to strain and dislocations for very thin films.

**Figure 7:**
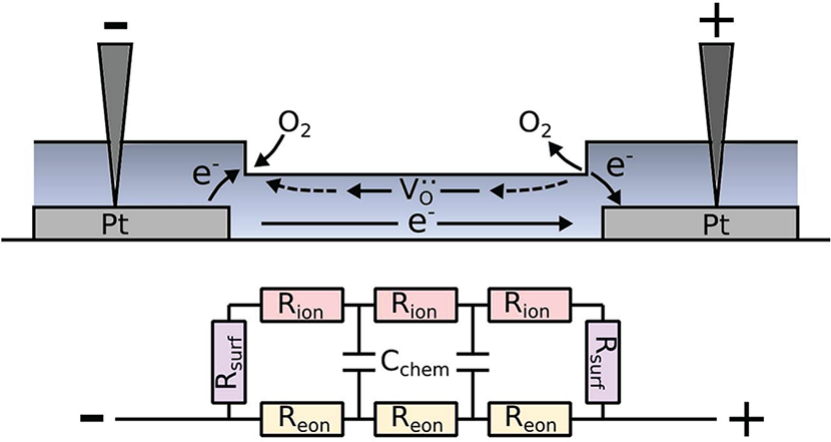
Sketch of the surface exchange reaction on the PCO10 surface facilitating an in-plane ionic conduction path between two Pt electrodes together with the corresponding equivalent circuit.

Moreover, a mid- to low-frequency feature is observed. For the case of ionically blocking contacts, this second feature would correspond to the transition from mixed to purely electronic conductivity.^45^ In the present case, however, surface oxygen exchange is possible on the PCO surface adjacent to the platinum fingers and the particularly clean conditions during *i*-PLD even enhance the reaction kinetics.^37^ Therefore, a parallel ionic conduction path is facilitated, with its relative importance controlled by the surface exchange resistance (this process is detailed in Figure 7). The size of this feature thus depends on the sheet resistance and the surface exchange resistance of PCO.

After a certain film thickness (≈60 nm), this second feature only very slightly decreases in size and its capacitive contribution increases linearly with thin film thickness. This is exactly what we would expect for a feature corresponding almost exclusively to the surface exchange resistance and the chemical capacitance of the film. The temperature control of this measurement is experimentally problematic. Reliable temperature measurement via pyrometer is not possible, as the mixture of MgO, STO, and Pt does not allow the conclusive evaluation of an emissivity. The temperature can, however, be estimated via the total PCO conductivity itself. According to literature, the conductivity in the plateau corresponds to a temperature of ≈720 °C.^46^

When the total conductivity extracted from the high-frequency feature reached a stable value, the surface was decorated with a total of 2 ML of SrO. As expected, the second feature decreased substantially in size, corresponding to the activation of the PCO10 surface by SrO. The total conductivity of the PCO10, however, was not affected by the decoration. Afterward the surface was restored by the deposition of PCO10 and the decoration experiment was repeated with SnO_2_. Again, as expected, the size of the second feature increases significantly, in line with the deactivation of the PCO10 surface by SnO_2_. Still, the total conductivity is unaffected by the decoration. While this result again disagrees with literature results on porous electrodes,^27^ it agrees very well with the XPS results of PCO10 thin films, which also do not show substantial changes of Pr oxidation states upon decoration. We therefore conclude that we do not observe large changes of the concentrations of electronic charge carriers on the surface of PCO10 after the decoration with binary oxides.

## Mechanistic Discussion

First, this study confirms the primary result of Nicollet et al.^27^ and substantiates the finding that the Smith acidity^28^ can be used as a qualitative descriptor for changes of the oxygen exchange kinetics of PCO10 electrodes. Acidic binary oxides relative to PCO10, like TiO_2_ and SnO_2_, decrease the oxygen exchange activity, while basic oxides relative to PCO10, such as SrO or CaO, accelerate the oxygen exchange kinetics. Second, the combination of *i*-PLD, LEIS, and NAP-XPS facilitates a detailed insight into the surface chemistry of decorated PCO10 and supplies a thorough experimental foundation for further discussions. Overall, the following experimental facts have to be considered:

- *i*-PLD experiments revealed that already submonolayer amounts of binary oxides have a strong impact on the surface exchange resistance of PCO10. While basic oxides (SrO, CaO) accelerate the oxygen exchange kinetics (a decrease down to 25% of the initial resistance), acidic oxides (SnO_2_, TiO_2_) lead to a strong increase of the surface exchange resistance (up to 600%).
- Although a significant change of the electrode impedance was observed after surface decoration, the same oxygen exchange mechanism seems to be active on both pure and decorated PCO10 electrodes, as the same activation energy and pO_2_ dependence of the polarization resistance were measured.
- NAP-XPS measurements revealed a change of the surface potential after decoration with binary oxides (full surface coverage has been secured by LEIS measurements). While a more negatively charged surface was observed on electrodes decorated with SnO_2_, decoration with SrO led to more positively charged surface.
- NAP-XPS measurements did not detect major changes of the electron concentration (Pr^3+^) on the surface of PCO10 after decoration with binary oxides.
- In-plane conductivity measurements support the conclusion that the decorations do not cause substantial changes of electron concentrations on decorated PCO10 surfaces.

**Figure 8:**
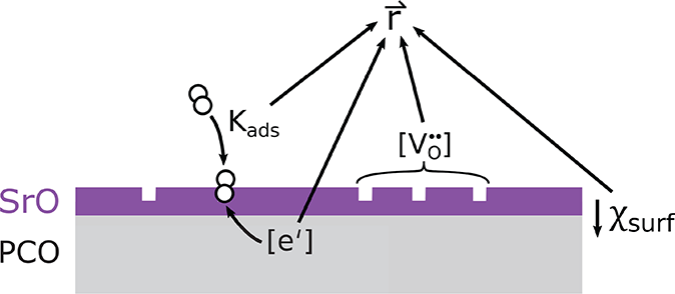
Surface decorations (here SrO) might affect different contributions to the oxygen exchange reaction rate: molecular O_2_ adsorption equilibria, surface oxygen vacancy concentrations, electronic charge carrier concentrations in the subsurface, and any existing surface potential step.

In literature, a variety of explanations have been brought forward concerning the underlying mechanism of the kinetic effect of surface decorations. These range from an altered concentration of active sites^29,47^ over the formation of active zones between two phases^48^ to changes of defect concentrations and adsorption behavior.^27,49^ The results presented here suggest that changing defect concentrations induced by surface band bending are not the cause for the altered oxygen exchange kinetics of decorated surfaces and that a further parameter has to be introduced into the discussion, the surface potential step. In the following, we will discuss several fundamental aspects of the oxygen exchange reaction and how surface decorations might affect these aspects. We propose an interplay of different processes which all affect the oxygen exchange reaction rate and which are visualized in Figure 8.

From the results of our NAP-XPS measurements we have concluded that decoration with binary oxides significantly modifies the surface potential, requiring charge transfer from or to the surface decoration. According to the direction of the surface potential change, we find that acidic decorations lead to a negative surface charge and basic decorations to a positive surface charge. This is also in line with the common definitions of acidity, where bases usually act as the donor of negative charge. In Smith’s definition of acidity,^28^ a basic oxide tends to transfer O^2–^ ions to oxides of higher acidity (as is the case for SrO on PCO). The thereby established electric field at the surface will affect the reaction rate directly if charge is transferred across this field during the oxygen exchange reaction. However, as Smith’s acidity definition is only a simplified model for ionic charge transfer between binary oxides, it is necessary to discuss how such redistribution processes could proceed in mixed conducting oxides.

Mobile charge carriers in PCO are generally either electrons (primarily Pr^3+^) or oxygen vacancies, V_O_^··^. As space charges in mixed ionic and electronic conductors are commonly related to chemical potential differences,^50^ the exact redistribution of charge strongly depends both on how a decoration layer affects the chemical potentials of oxygen vacancies and electrons in the host material and on the chemical potentials of these species in the decoration layer itself. It is likely, that a usually insulating material such as SrO can form oxygen vacancies and partly covalent bonds at an interface with a mixed conducting material. Hence, the evolution of charge carrier concentrations in such heterointerfaces is highly nontrivial and requires further computational investigation, going beyond the scope of this study. At this point, we also want to refer to a recent study on the effects of acidic adsorbates on LSC surfaces, combining experimental and computational approaches.^51^ Similar to acidic decorations, acidic adsorbates cause a significantly increased surface exchange resistance as well as an increased work function (negative surface charge) and charge redistribution in this system has largely been associated with oxygen atoms and charge transfer toward the adsorbate.

Indicated by the formation of a positive surface charge for basic decorations (negative for acidic decorations), we can further assume that basic decorations promote the formation of positively charged oxygen vacancies, which have been proposed to be a decisive factor for fast oxygen exchange kinetics.^52−55^ This hypothesis is also supported by the lower binding energy O 1s species measured on SrO-decorated PCO10 (and *vice versa* for SnO_2_-decorated PCO10, see Figure 4C). Nevertheless, independent of the exact nature of charge redistribution processes, it is evident that defect concentrations in the surface and subsurface will change upon decoration, altering oxygen exchange reaction rates, which contain contributions of both vacancy and electron concentrations.

As a last noteworthy aspect, we suspect that surface decorations also influence reaction energetics such as adsorption equilibria,^49^ further complicating the situation. This claim is again supported by recent computational results on acidic adsorbates on LSC,^51^ where calculations revealed that acidic adsorbates cause strongly increased adsorption barriers for O_2_ molecules and render O_2_ adsorbates in surface vacancies energetically unfavorable.

In summary, we believe that a convolution of several effects is responsible for the observed change of the OER kinetics on decorated PCO electrodes. As they potentially partly counterbalance each other, it is not straightforward to disentangle the contributions of each effect. To gain conclusive insight into the defect chemistry of decorated surfaces, detailed computational studies of energetics and charge redistribution at decorated surfaces are necessary. Nevertheless, as has been shown in this study, surface decorations represent a promising approach to enhance the catalytic activity of MIEC surfaces toward oxygen exchange and this study lays the foundations for a more comprehensive investigation of the fundamental processes responsible for the effect of surface decorations on the oxygen exchange activity.

## Conclusion

PCO10 thin film electrodes were decorated with up to 1.5 (nominal) monolayers of binary oxides with different Smith acidity (SrO, CaO, TiO_2_, and SnO_2_ from basic to acidic). The effect of surface decorations on the OER resistance was tracked by *i*-PLD impedance spectroscopy which enabled very precise measurements of the OER activity. These experiments confirmed previous results that the Smith acidity of MIEC surfaces is a sensitive descriptor of the oxygen exchange activity (i.e., basic oxides increase the OER kinetics and acidic oxides have the opposite effect), while the mechanism of the oxygen exchange reaction itself seems to be unaffected. Despite substantial changes of the surface exchange resistance, the pO_2_ and temperature dependence remained unchanged upon surface decoration. Changes of the surface chemistry and the surface potential were investigated by NAP-XPS and the measurements revealed that surface decorations substantially alter the surface potential according to their acidity (acidic decorations lead to a more negative surface charge and *vice versa*). However, defect concentrations in the near surface region do not follow a corresponding space charge model, indicating more complex interactions at the surface. Based on these results, we discuss the oxygen exchange reaction on decorated surfaces, outlining several different effects that surface decorations might have on a PCO electrode and its oxygen exchange kinetics.

## Methods

### Experimental Methods

**Figure 9:**
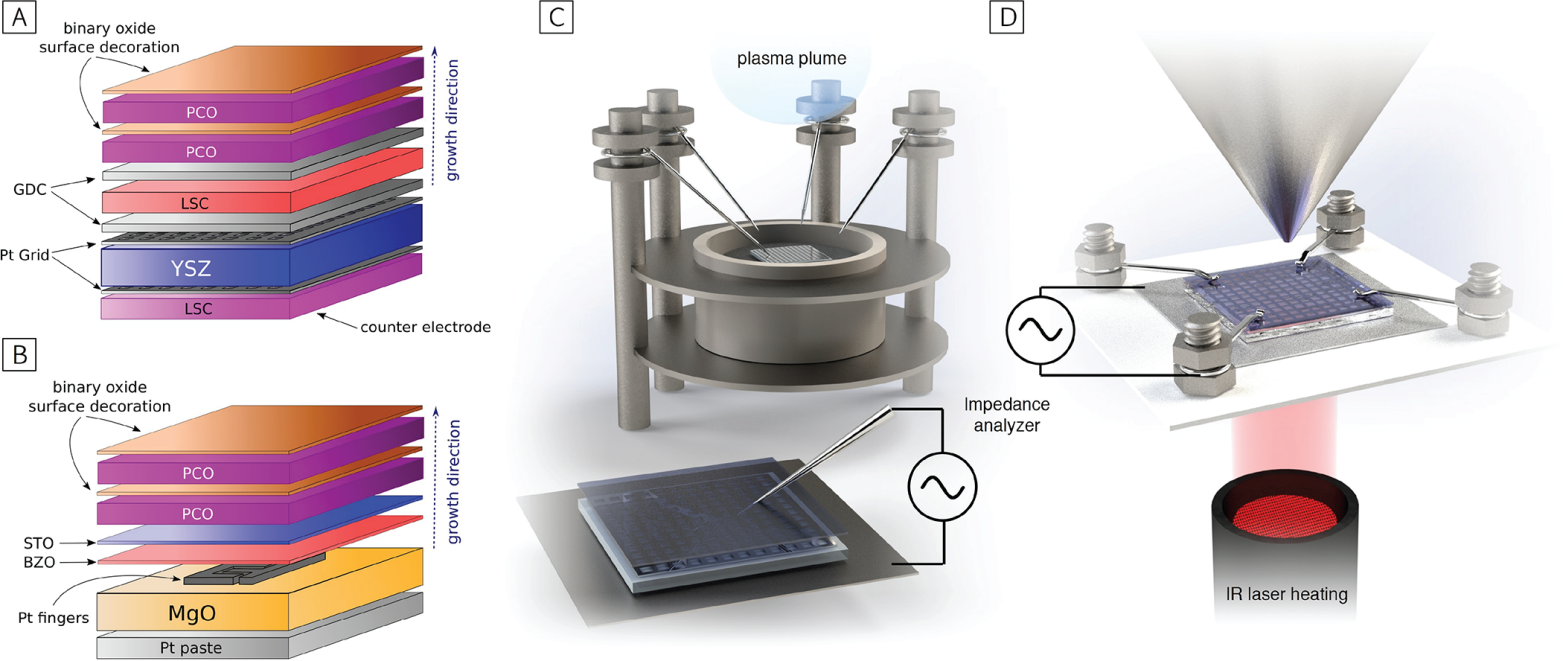
(A) Sketch of the multilayer sample used during cross-plane *i*-PLD measurements. (B) Sketch of the multilayer sample used during in-plane *i*-PLD measurements. (C) *i*-PLD setup sketch. (D) NAP-XPS setup sketch.

(001)-oriented yttria stabilized zirconia (YSZ, 9.5 mol % Y_2_O_3_, Crystec GmbH, Germany) single crystals (5 × 5 × 0.5 mm^3^) were used as substrates for all across-plane *i*-PLD measurements (Figure 9A). Ti/Pt grids (15/5 μm holes/mesh, 5/300 nm Ti/Pt thickness) were prepared on both sides of the substrates by lift-off photolithography and magnetron sputtering. As a counter electrode, nanoporous LSC was deposited on top of one of the Ti/Pt grids via PLD at 450 °C, in 0.4 mbar O_2_, at a substrate-target distance of 5.0 cm and a laser frequency of 5 Hz.^23,56^ On the WE side, a multilayer structure of Gd_0.2_Ce_0.8_O_2−δ_ (GDC, 5 nm), LSC (50 nm), and GDC (5 nm) was deposited on the platinum grid structure at a temperature of 600 °C and 0.04 mbar O_2_. The multilayer structure was applied to ensure sufficient in-plane conductivity for the PCO10 thin film grown on top of it, while still enabling subsequent epitaxial growth of the fluorite PCO on the perovskite LSC.

During *i*-PLD measurements, PCO10 was grown on top of the GDC|LSC|GDC multilayer structure via PLD at a temperature of 600 °C, a pressure of 0.04 mbar O_2_, a substrate-target distance of 6.0 cm and a laser frequency of 2 Hz. For all depositions, a KrF excimer laser (λ = 248 nm, Lambda Physics, COMPex Pro 201) with a laser fluence of ∼1.1 J/cm^2^ at the target was used. For the decoration of the PCO10 thin film electrodes, targets of the respective binary oxides (SrO, CaO, TiO_2_, SnO_2_) were prepared from the respective calcined powders (Sigma-Aldrich, >99.9%) by isostatic pressing and sintering. Basic oxides had to be calcined before pressing (12 h at 1200 °C in O_2_ flow). During the period of the measurements, targets were stored in a desiccator with a drying agent (silica gel) under vacuum to avoid hydration of the oxides. Before every *i*-PLD measurement, the deposition rate of each decoration target was determined with a quartz balance inside the PLD chamber at 0.04 mbar O_2_ and room temperature. Usually, amounts corresponding to up to 1.5 nominal monolayers of the respective binary oxide were deposited on the PCO10 electrode (see Table 1).

**Table 1:** Deposition Parameters of the Binary Oxides^a^

material	deposited monolayers	nominal thickness (nm)	deposited mass (ng/mm^2^)
CaO	1.5	0.37	0.40
SrO	1.5	0.38	0.61
TiO_2_	1.4	0.33	0.53
SnO_2_	1.3	0.31	0.86

a Lattice parameters from literature^57−60^ have been used to calculate the necessary amount of material to deposit 1.5 ML.

Impedance spectroscopic measurements inside the PLD chamber were performed on a custom-made heating stage^61^ with an Alpha-A High Performance Frequency Analyzer and Electrochemical Test Station POT/GAL 30 V/2A setup (Novocontrol Technologies, Germany) in a frequency range from 10^6^ to 10^–1^ Hz. For a more detailed description of *i*-PLD measurements, the reader is referred to earlier studies employing this technique.^20,29^ The sample temperature was measured via the high-frequency intercept of the recorded impedance spectra, consisting of the only slightly temperature-dependent resistances of the setup and the Ti/Pt grid and the strongly temperature-dependent electrolyte resistance.^32,62^

In addition, a novel measurement technique was employed during *i*-PLD, allowing the tracking of the total in-plane conductivity of a growing and decorated PCO10 thin film. Interdigitating finger electrodes were prepared on MgO single crystals (Crystec GmbH, Germany) by Pt sputtering, photolithography, and ion beam etching (Figure 9B).^63^ To warrant epitaxial growth of PCO10 on MgO, a buffer layer structure of 5 nm BaZrO_3_ (BZO) and 5 nm SrTiO_3_ (STO) was deposited prior to PCO10 growth.^64^ The interdigitating structures yielded 10 μm wide fingers with a 100 μm spacing and a total meander length of 2.43 cm. On these samples, *i*-PLD was used to grow a PCO10 thin film and to decorate the surface with SrO and SnO_2_ while tracking the in-plane impedance response.

XPS spectra were acquired in a lab-based NAP-XPS setup with a PHOIBOS NAP photoelectron analyzer (SPECS, Germany) and a monochromated Al K-alpha XR 50 MF (microfocus) X-ray source. Therein, the solid oxide cell was mounted on a special sample holder with a centered 4.5 × 4.5 mm^2^ sized hole for heating with a near-IR diode laser.^65^ Electrical contacts for working and counter electrodes were established by Pt–Ir wires and tips, respectively. The temperature of the sample was controlled via the conductivity of the YSZ electrolyte that was measured *in situ* by EIS. AP-XPS measurements were conducted at 1 mbar O_2_ and an electrode temperature of 550 °C. XPS spectra were collected at an analyzer pass energy of 30 eV, which provided a reasonable balance of count rate and energy resolution.

The outermost atomic layers of decorated PCO10 surfaces were investigated with LEIS, using a QTAC 100 LEIS system (IONTOF GmbH, Germany). A 5 keV ^20^Ne^+^ primary analysis beam at a 90° incidence angle was used to measure cation signals at an analyzed area of 1 × 1 mm^2^ and a beam current of 1 nA. For depth profiling, a 500 eV ^40^Ar^+^ beam with a beam current of 100 nA was used. Before analysis, the samples were cleaned by reactive oxygen cleaning in a preparation chamber.

## Supporting Information Available

X-ray diffraction data of epitaxial multilayer structures, LEIS depth profiles of decorated PCO surfaces (PDF)
