# Supplementary material for: Surface Decorations on Mixed Ionic and Electronic Conductors: Effects on Surface Potential, Defects, and the Oxygen Exchange Kinetics
Source: ACS Appl Mater Interfaces. 2023 May 22;15(22):26787–98. doi: 10.1021/acsami.3c03952 (PMC10251416; doi:10.1021/acsami.3c03952)
Supplement: Supplementary file 1 — am3c03952_si_001.pdf [file am3c03952_si_001.pdf]

.

Supporting Information: Surface decorations on mixed ionic and electronic conductors: effects on surface potential, defects and the oxygen exchange kinetics

Christoph Riedl†[1] Matthäus Siebenhofer†[1,2], Andreas Nenning[1], George E. Wilson [3], John Kilner [3], Christoph Rameshan[4], Andreas Limbeck[1], Alexander Karl Opitz[1], Markus Kubicek[1], Juergen Fleig[1]*

* corresponding author
E-mail: matthaeus.siebenhofer@tuwien.ac.at

[1] Institute of Chemical Technologies and Analytics
TU Wien
Getreidemarkt 9-E164, 1060 Vienna, Austria

[2] CEST Kompetenzzentrum für elektrochemische Oberflächentechnologie GmbH
TFZ – Wiener Neustadt Viktor-Kaplan-Strasse 2
2700 Wiener Neustadt, Austria

[3] Department of Materials
Imperial College
Exhibition Road, London SW7 2BX , United Kingdom

[4] Chair of Physical Chemistry
Montanuniversität Leoben
8700 Leoben, Austria

1 X-ray diffraction measurements on epitaxial PCO thin films

Fig. 1 a) shows a θ-2θ scan of the multilayer system used in th i-PLD studies. A GDC/LSC/GDC system is deposited on the YSZ single crystal to ensure sufficient in-plane conductivity and PCO10 is deposited on top of the multilayer. As can be seen in the diffractogram, PCO is oriented purely in the [001] direction, the same holds for LSC. The GDC signal is hidden between the YSZ and PCO peaks. Slight Pt remnants on the sample from a brushed Pt paste on the backside of the sample cause two additional minor reflexes.

For in-plane conductivity measurements, the abovementioned multilayer structure is not suitable because the YSZ single crystal as well as the multilayer system would dominate all in-plane conduction. Instead, PCO10 is deposited on a MgO single crystal with a BZO/STO buffer layer, ensuring (001) oriented growth. The corresponding diffractogram is shown in Fig. 1 b). Again PCO is purely [001] oriented and a slight reflex corresponding to the (002) reflex of the BZO/STO layer is visible.

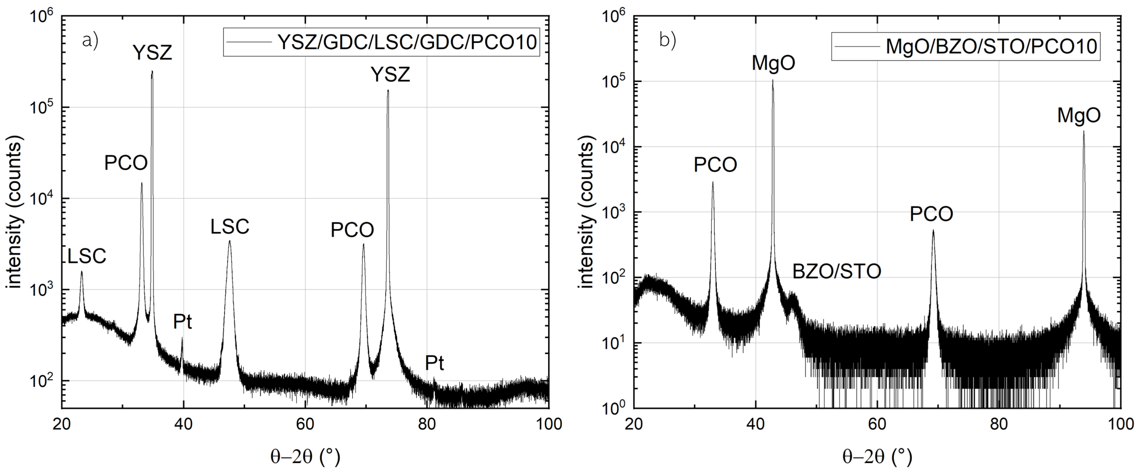

Figure S 1: a) Diffractogram of a YSZ/GDC/LSC/GDC/PCO10 multilayer with all assigned reflexes; b) Diffractogram of an MgO/BZO/STO/PCO10 multilayer with all assigned reflexes.

2 Low energy ion scattering depth profiles

Depth profiles of PCO thin films decorated with 2 ML SrO and SnO_2 were recorded with LEIS to check the distribution of decoration cations in the PCO thin film. Fig. 2 A) and B) show selected LEIS spectra during the depth profiles at the outermost surface (A) and at the end of the depth profile (B).

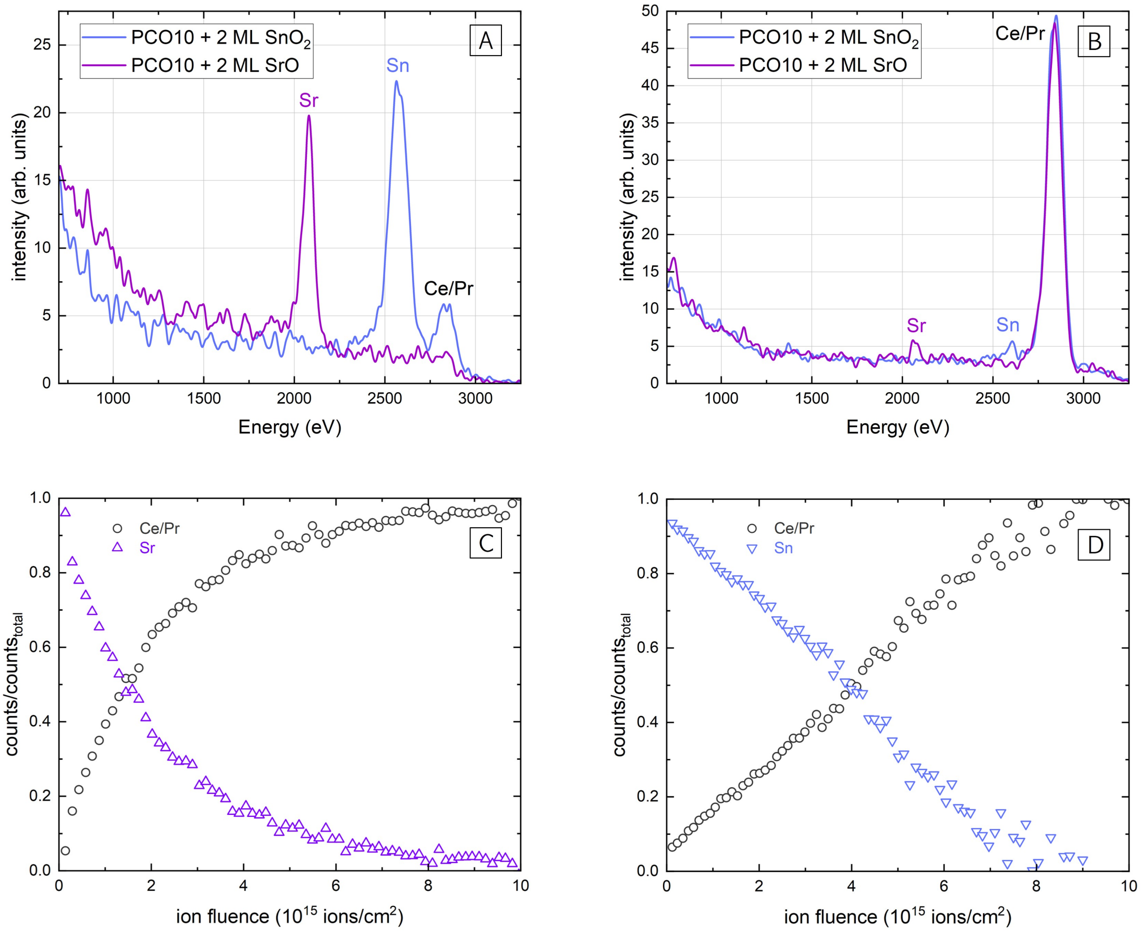

Figure S 2: A) LEIS spectra of the outermost surface of Sr- and Sn-decorated PCO10; B) LEIS spectra in the bulk of Sr- and Sn-decorated PCO10; C) depth profile of a Sr-decorated PCO10 thin film; D) depth profile of a Sn-decorated PCO10 thin film. All thin films were decorated with 2 ML of the respective binary oxide.
